# Cold-aggravated pain in humans caused by a hyperactive Na_V_1.9 channel mutant

**DOI:** 10.1038/ncomms10049

**Published:** 2015-12-08

**Authors:** Enrico Leipold, Andrea Hanson-Kahn, Miya Frick, Ping Gong, Jonathan A. Bernstein, Martin Voigt, Istvan Katona, R. Oliver Goral, Janine Altmüller, Peter Nürnberg, Joachim Weis, Christian A. Hübner, Stefan H. Heinemann, Ingo Kurth

**Affiliations:** 1 Department of Biophysics, Center for Molecular Biomedicine, Friedrich Schiller University Jena & Jena University Hospital, 07745 Jena, Germany; 2 Department of Pediatrics, Stanford University School of Medicine, Stanford, California 94305-5208, USA; 3 Department of Genetics, Stanford University School of Medicine, Stanford California 94305-5208, USA; 4 Institute of Human Genetics, Jena University Hospital, 07743 Jena, Germany; 5 Institute of Neuropathology, RWTH Aachen University Hospital, 52074 Aachen, Germany; 6 Cologne Center for Genomics (CCG), University of Cologne, 50931 Cologne, Germany; 7 Institute of Human Genetics, University of Cologne, 50931 Cologne, Germany; 8 Cologne Excellence Cluster on Cellular Stress Responses in Aging-Associated Diseases (CECAD), University of Cologne, 50931 Cologne, Germany; 9 Center for Molecular Medicine Cologne (CMMC), University of Cologne, 50931 Cologne, Germany

## Abstract

Gain-of-function mutations in the human *SCN11A*-encoded voltage-gated Na^+^ channel Na_V_1.9 cause severe pain disorders ranging from neuropathic pain to congenital pain insensitivity. However, the entire spectrum of the Na_V_1.9 diseases has yet to be defined. Applying whole-exome sequencing we here identify a missense change (p.V1184A) in Na_V_1.9, which leads to cold-aggravated peripheral pain in humans. Electrophysiological analysis reveals that p.V1184A shifts the voltage dependence of channel opening to hyperpolarized potentials thereby conferring gain-of-function characteristics to Na_V_1.9. Mutated channels diminish the resting membrane potential of mouse primary sensory neurons and cause cold-resistant hyperexcitability of nociceptors, suggesting a mechanistic basis for the temperature dependence of the pain phenotype. On the basis of direct comparison of the mutations linked to either cold-aggravated pain or pain insensitivity, we propose a model in which the physiological consequence of a mutation, that is, augmented versus absent pain, is critically dependent on the type of Na_V_1.9 hyperactivity.

## Abstract (web summary)

A mutation in the sodium channel Nav1.9 has been identified in a family and shown to associate with cold-aggravated pain. Here, the authors characterize the electrophysiological consequences of this mutation and propose a mechanism for the pain that the individuals experience.

## Graphical abstract

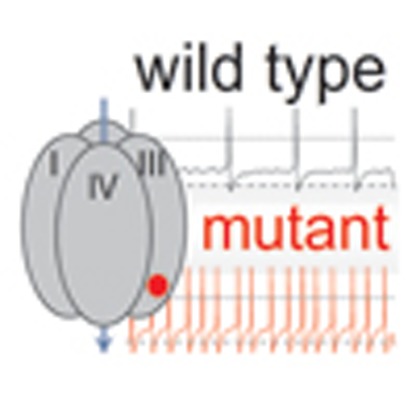

Voltage-gated Na^+^ (Na_V_) channels of peripheral afferents are essential for pain perception because they initiate action potentials in nociceptive nerve fibres. The family of mammalian Na_V_ channels consists of nine members (Na_V_1.1–1.9) of which Na_V_1.7, Na_V_1.8 and Na_V_1.9 are the most abundant isoforms in nociceptive neurons^1^ of dorsal root ganglia (DRG). While Na_V_1.7- and Na_V_1.8-mediated currents are the main contributors to the fast action potential upstroke in nociceptors, Na_V_1.9 channels modulate the threshold of nociceptor excitability by influencing the resting membrane potential (RMP)^2^,^3^,^4^.

Several monogenic human pain disorders are linked to altered Na_V_ channel function^5^. For example, biallelic loss-of-function mutations in *SCN9A*, encoding Na_V_1.7, result in inability to experience pain by impairing the electrical signalling of nociceptors^6^,^7^. In contrast, gain-of-function mutations in *SCN9A* result in hyperexcitability of nociceptors and cause debilitating pain disorders such as primary erythromelalgia and paroxysmal extreme pain disorder^8^,^9^. Notably, environmental warmth aggravates pain in primary erythromelalgia patients while cooling of affected extremities relieves pain, suggesting a marked temperature dependence of the phenotype^10^,^11^. More recently, Na_V_1.7 variants with increased activity have been associated with paroxysmal itch^12^ and painful degeneration of sensory nerve terminals (small-fiber neuropathy)^13^. Painful neuropathies have also been linked to gain-of-function variants of Na_V_1.8, encoded by *SCN10A* (ref. 14).

The role of Na_V_1.9 in human pain perception is less clear and has only recently begun to be elucidated. The first pathogenic mutation identified in human *SCN11A*, the gene encoding Na_V_1.9, was the heterozygous *de novo* mutation p.L811P, which confers gain-of-function properties to Na_V_1.9 and causes congenital insensitivity to pain^15^,^16^. Further studies have linked gain-of-function mutations in Na_V_1.9 to familial episodic pain and painful neuropathy^17^,^18^,^19^. At present it is not clear how the gain-of-function of Na_V_1.9 channels can result in such opposing pain phenotypes.

We here describe a Na_V_1.9 mutation (p.V1184A) that causes early onset cold-aggravated familial episodic pain. Electrophysiological evaluation reveals that the mutation enhances the activity of Na_V_1.9 by left shifting the voltage dependence of channel opening, thereby giving rise to hyperexcitability of nociceptors. In line with the temperature sensitivity of the patients' phenotype, p.V1184A-dependent nociceptor excitability is less attenuated by cold than the excitability of wild-type neurons, suggesting a temperature-dependent contribution of the novel Na_V_1.9 variant to nociceptor function.

## Results

### Clinical description and whole-exome sequencing

**Figure 1:**
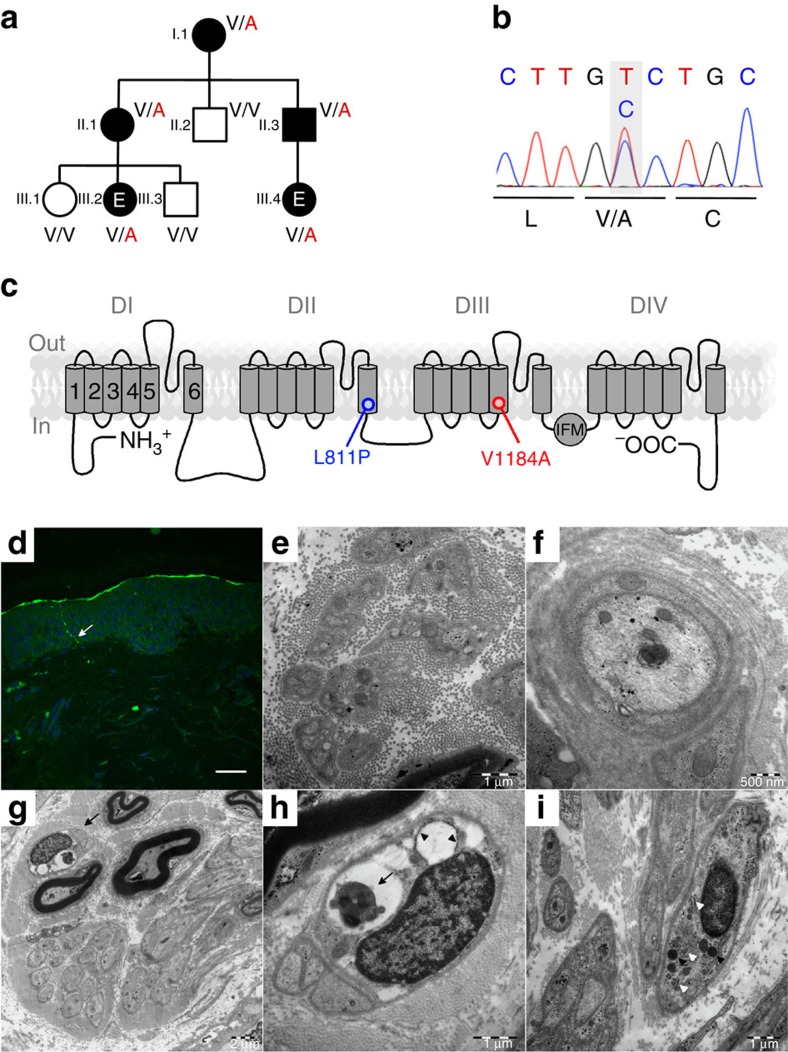
A heterozygous mutation in *SCN11A* in patients with cold-induced episodic pain. (**a**) Pedigree of the family with episodic pain. Genotype of the p.V1184A mutation is indicated. V/A=heterozygous mutation carrier, V/V=wild type. Whole-exome sequencing was performed in individuals III.2 and III.4 (marked with ‘E') (**b**) Confirmation of the *SCN11A* mutation by Sanger sequencing. (**c**) Membrane topology of the α subunit of Na_V_1.9, encoded by *SCN11A*. The four homologous domains (DI-DIV) are indicated; position of mutations p.L811P (blue) causing pain insensitivity and p.V1184A (red) causing cold-aggravated peripheral pain is highlighted. (**d**) Reduced epidermal nerve fiber density in the father of the patient. Arrow: PGP9.5-immunoreactive epidermal nerve fiber. (Scale bar, 20 μm). (**e**) Stacks of Schwann cell processes containing only few unmyelinated axons. (Scale bar, 1 μm). (**f**) Autophagic vacuole in an unmyelinated axon. (Scale bar, 500 nm). (**g**) Small dermal nerve fascicle containing myelinated and unmyelinated axons. Arrow: abnormal vacuoles in an endoneurial Schwann cell. (Scale bar, 2 μm). (**h**) At higher magnification, autophagic material is found in one of the vacuoles (arrow). Another vacuole (arrowheads) is lined by ribosomes and connected to the nuclear envelope. (Scale bar, 1 μm). (**i**) Prominent rough endoplasmic reticulum, multivesicular bodies (white arrowheads) and prominent osmiophilic structures, probably lysosomes (black arrowheads) in an endoneurial Schwann cell. (Scale bar, 1 μm).

We studied a three-generation family of mixed European ancestry with severe episodic pain of unclear aetiology (Fig. 1a and Table 1). Onset of chronic pain in the 6-year-old female proband III.4 was within the first year of life. Pain comes on quickly and lasts for about 20–30 min. Reported triggers of pain are gluten and notably low ambient temperature. Pain usually starts in the joints and radiates to the arms and legs. Occasionally, it is accompanied by flushing of the neck and face. The lower extremities are primarily affected but her upper extremities can also be symptomatic. Pain episodes most commonly occur in the late afternoon or early evening. Her symptoms have responded to ibuprofen, colchicine and naproxen. Her motor milestones and intellectual development are typical. Her father (II.3), paternal aunt (II.1), paternal aunt's daughter (III.2) and paternal grandmother (I.1) had similar symptoms suggesting an autosomal-dominant disorder underlying the pain phenotype. The patient's father's pain episodes were first noticed at ∼18 months of age and were associated with flushing of his neck and chest when he was younger. His pain originates in his joints, radiates distally and primarily affects his lower extremities. Less often the upper extremities may be involved. The sensation is described as feeling ‘on fire'. The duration of episodes is between 20 and 30 min during which fine motor ability may be impaired and ambulation is uncomfortable. The frequency of pain episodes is typically two to three times per month when controlled with ibuprofen and a gluten-free diet. Episodes may occur any hour but most often begin at night and may also be provoked by stressors such as exhaustion and illness. The proband's father also reports frequent constipation since the age of 18. The proband's grandmother's symptoms reportedly began in early infancy and similarly occur in the lower extremities and on occasion the upper extremities. Her pain also begins in the joints and radiates outward. Affected joints are limited to the wrists, elbows, knees and ankles. She reports taking ibuprofen prophylactically and avoiding gluten as a means to reduce the number of episodes to a frequency of two to three times per month. In the absence of medication, the episodes last ∼3 h consisting of alternating cycles of pain and no pain in intervals of 20 min. The pain impairs her fine motor manipulation, makes ambulation uncomfortable and can be partially alleviated by positional changes. She has had gastrointestinal symptoms including episodic constipation and diarrhoea since the age of 18. The proband's paternal aunt and paternal first cousin also experience similar pain episodes but of lesser severity. Similar to other family members, the pain originates in their joints and radiates distally. Non-steroidal anti-inflammatories have reduced the frequency of pain episodes. The aunt uses ibuprofen and the cousin uses ibuprofen and naproxen. The aunt's pain is somewhat eased by warmth but this is not the case for other affected family members. Of note, cold ambient temperatures can trigger episodes in the aunt. There have been no appreciable developmental or neurologic deficits.

Quantitative evaluation of skin biopsies stained for PGP9.5 revealed a prominent decrease of the intra-epidermal nerve fiber density in the skin biopsies of the father (II.3; Fig. 1d), of the paternal aunt (II.1) and of the grandmother (I.1), compared with published reference values^20^, indicating a small-fiber neuropathy (6.0, 7.4 and 8.8 nerve fibres/mm, respectively, compared with the reference value of 17.07±6.51 nerve fibres/mm (ref. 20)). Electron microscopy of dermal nerve fascicles demonstrated stacking of Schwann cell processes indicative for a loss of unmyelinated axons (Fig. 1e) and signs of altered endoplasmic reticulum and lysosomal function as well as autophagy in axons and Schwann cells (Fig. 1f–i). Whole-exome sequencing was performed in the index case and her affected cousin and the data were filtered for shared rare variants that were absent from dbSNP, 1000-Genomes project, the Exome Variant Server, or had a low prevalence in the ExAC browser. No causal mutation was identified in known genes involved in episodic pain syndromes (*SCN9A*, *SCN10A* and *TRPA1*). Of the rare heterozygous genetic variants shared by both individuals, a missense change in *SCN11A*, c.3551T>C, p.V1184A (chr3:38913144A>G, hg19), was the most likely candidate to cause the disorder. The variant was confirmed by Sanger sequencing (Fig. 1b), and segregation with the pain phenotype was verified by genotyping of available family members (Fig. 1a). The variant affects a highly conserved amino-acid residue in the S5 segment in domain III of Na_V_1.9 (Fig. 1c).

### Assessment of p.V1184A in voltage-clamp experiments

To analyse the consequences of mutation p.V1184A for the function of Na_V_1.9, we performed whole-cell voltage-clamp experiments in ND7/23 cells heterologously expressing either human wild-type Na_V_1.9 or Na_V_1.9-V1184A mutant channels. All recordings were carried out at 30 °C, which is close to peripheral skin temperature^21^,^22^. To assess the temperature sensitivity of channel function, data were furthermore collected at 20 °C, a temperature selected to simulate cooling that triggers painful episodes in the proband and her aunt.

**Figure 2:**
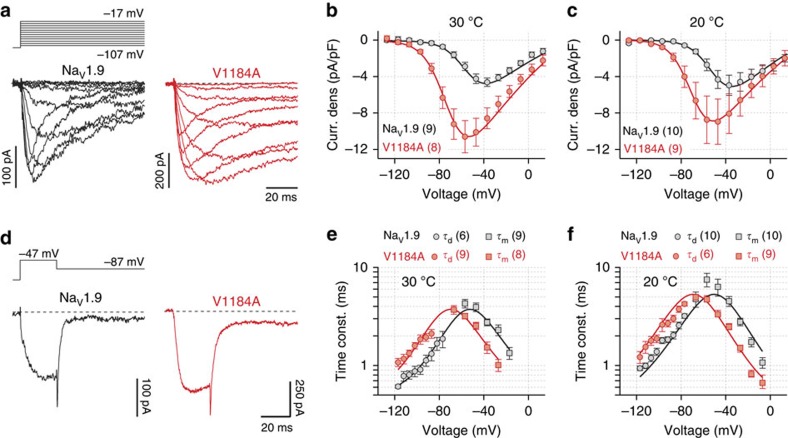
Mutation p.V1184A enhances activation of Na_V_1.9. (**a**) Representative whole-cell current traces recorded at 20 °C from a ND7/23 cell transiently expressing Na_V_1.9 (black) or Na_V_1.9-V1184A (red) channels in response to depolarizing voltages ranging from –107 to –17 mV in steps of 10 mV. (**b**,**c**) Mean current densities of ND7/23 cells expressing either Na_V_1.9 (black) or Na_V_1.9-V1184A (red) measured at 30 °C (**b**) or 20 °C (**c**) as a function of voltage. Superimposed fits describe the voltage dependence of channel activation according to equation (1). (**d**) Representative whole-cell tail currents recorded at 20 °C from a ND7/23 cell transiently expressing Na_V_1.9 (black) or Na_V_1.9-V1184A (red) channels in response to a hyperpolarizing voltage step from –47 to –87 mV. (**e**,**f**) Single-exponential time constants of channel activation (*τ*_m_, squares) and deactivation (*τ*_d_, circles) obtained at 30 °C (**e**) or 20 °C (**f**) from experiments as shown in **a** and **d**, plotted as function of voltage. Superimposed curves are data fits according to equation (5) characterizing the voltage dependence of activation and deactivation kinetics of Na_V_1.9 and Na_V_1.9-V1184A. Data points represent mean values with numbers of experimental replicates indicated in parentheses. Error bars in all plots represent s.e.m.

Both, wild-type and mutant channels gave rise to currents with slow activation and inactivation kinetics typical for Na_V_1.9 (Fig. 2a). Compared with the wild type, Na_V_1.9-V1184A channels yielded larger current densities particularly at low voltages, being significant between −97 and −57 mV (*P*<0.05, two-tailed *t*-test) at both temperatures tested (Fig. 2b,c). Mean current densities of wild-type and mutant channels showed no significant temperature dependence in the entire voltage range analysed (*P*>0.05 in all cases, two-tailed *t*-test). However, at both temperatures the larger current densities of cells expressing mutant p.V1184A were accompanied by three major effects: mutated channels displayed (i) a strongly left-shifted voltage dependence of channel activation, effectively increasing Na^+^ influx at resting membrane voltages (Fig. 2b,c), (ii) accelerated channel opening kinetics (Fig. 2e,f) and (iii) slowed channel closing kinetics (Fig. 2d). Analysis of the voltage dependence of peak current amplitudes (Fig. 2b,c) and the voltage dependence of channel opening and closure kinetics (Fig. 2e,f) according to a one-step gating scheme (equations (1) and (5)) revealed that the mutation shifted the half-maximal voltage for channel activation (*V*_m_) from −52.0±2.6 to −69.5±2.9 mV at 30 °C (*P*<0.001, two-tailed *t*-test) and from −47.5±2.5 to −67.0±2.7 mV at 20 °C (*P*<0.001, two-tailed *t*-test). The associated slope factors (*k*_m_), which reflect the voltage dependence of channel activation, were not affected by the mutation (9.1±0.7 and 10.1±0.8 mV for Na_V_1.9 at 30 and 20 °C; 9.8±1.0 and 9.8±0.7 mV for p.V1184A at 30 and 20 °C, respectively; both *P*>0.05, two-tailed *t*-test). The voltage dependence of opening and closing kinetics were compatible with a gating charge transfer of 2.8±0.5 e_0_ and 2.6±0.3 e_0_ and associated symmetry factors specifying the charge fraction assigned to channel opening of 0.53±0.08 and 0.47±0.02 at 30 and 20 °C, respectively. The Q_10_ temperature coefficients of the channel's opening kinetics, *τ*_a_ (1.50±0.11 for Na_V_1.9 between −57 and −17 mV; 1.45±0.06 for p.V1184A between −67 and −27 mV), and the closing kinetics, *τ*_d_ (1.49±0.07 for Na_V_1.9 between −117 and −77 mV; 1.31±0.04 for p.V1184A between −117 and −87 mV), were not markedly affected by the mutation (*P*>0.05, two-tailed *t*-test), suggesting that temperature changes affect opening and closing of wild-type and mutant channels in a similar manner.

**Figure 3:**
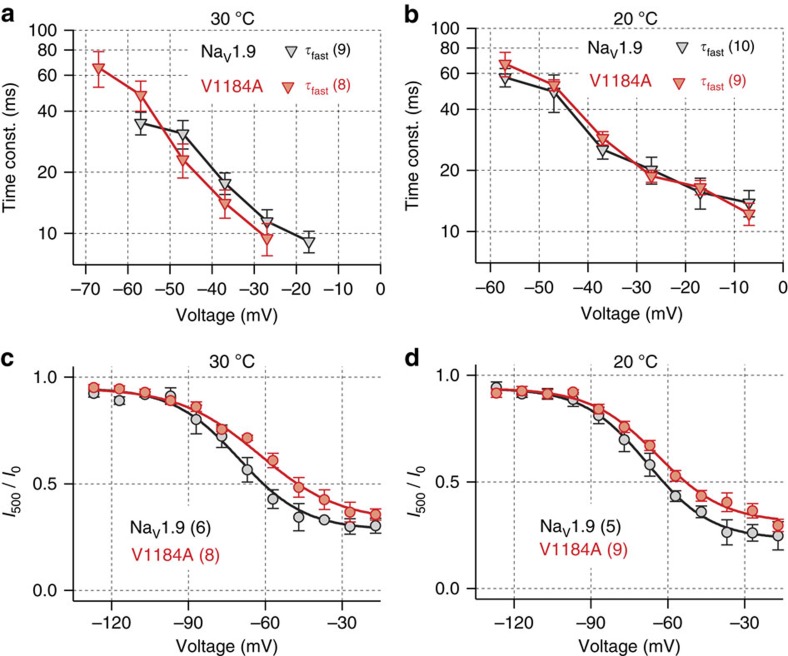
Effects of mutation p.V1184A on inactivation properties of Na_V_1.9 channels. (**a**,**b**) Single-exponential time constants of fast inactivation of Na_V_1.9 (black) and Na_V_1.9-V1184A (red) channels obtained from experiments as shown in Fig. 2a at 30 °C (**a**) or 20 °C (**b**) plotted as a function of voltage. Straight lines connect data points for clarity. (**c**,**d**) Voltage dependence of steady-state fast inactivation of Na_V_1.9 (black) and Na_V_1.9-V1184A (red) channels at 30 °C (**c**) or 20 °C (**d**), analysed according to equation (3). Data points represent mean values with numbers of experimental replicates indicated in parentheses. Error bars in all plots represent s.e.m.

At either temperature, mutation p.V1184A did not affect the kinetics of fast channel inactivation (Figs 2a,b and 3a,b). Likewise, the Q_10_ values of the fast inactivation time constant, *τ*_fast_, were also not significantly different between wild-type and mutant channels (1.62±0.06 for Na_V_1.9 between −57 and −17 mV; 1.81±0.25 for p.V1184A between −67 and −27 mV). The half-maximal voltage of channel inactivation, *V*_h_, measured after 500 ms conditioning episodes at various voltages, was only marginally shifted in the mutant from −69.1±3.9 to −64.8±3.3 mV at 30 °C and from −71.5±3.5 to −65.6±1.2 mV at 20 °C, without reaching the level of significance (both *P*>0.05, two-tailed *t*-test). The associated slope factors (*k*_h_) of wild-type (−10.0±1.1 mV) and mutant channels (−11.2±0.8 mV) characterizing the voltage dependence of channel inactivation were not significantly different at 20 °C; however, at 30 °C the mutation decreased *k*_h_ from −9.3±1.2 to −12.8±1.0 mV (*P*<0.05, two-tailed *t*-test), thus effectively increasing the availability of mutated channels (Fig. 3c,d).

In summary, the mutation p.V1184A confers gain-of-function properties to Na_V_1.9 by enhancing channel opening at RMPs and increasing channel availability at both temperatures analysed.

### Influence of p.V1184A on DRG neuron excitability

**Figure 4:**
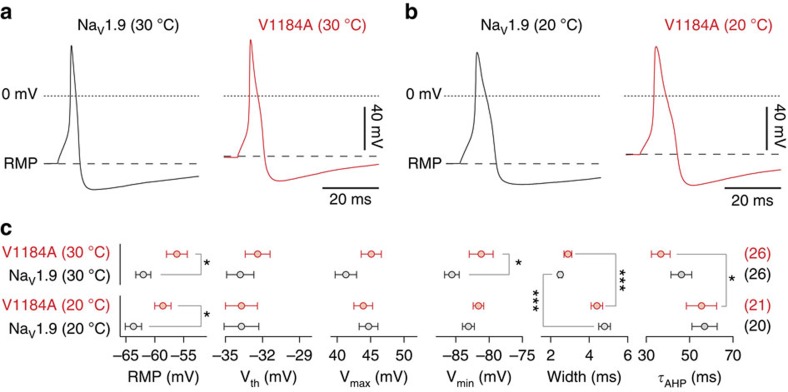
Na_V_1.9-V1184A channels depolarize DRG neurons. Representative single action potentials at 30 °C (**a**) or 20 °C (**b**), elicited in murine DRG neurons transfected with wild-type Na_V_1.9 (black) or mutant Na_V_1.9-V1184A (red) in response to current injections of 100–200 pA for 5 ms (**a**) or 10 ms (**b**). Dotted lines indicate 0 mV; dashed lines mark levels of the RMP. (**c**) Parameters characterizing action potential properties. *V*_th_: action potential voltage threshold, *V*_max_: maximum action potential voltage, *V*_min_: minimum voltage during after-hyperpolarization, width: action potential width at 0 mV, *τ*_AHP_: single-exponential time constant characterizing the relaxation of the membrane voltage from the action potential after-hyperpolarization (*V*_min_) back to the resting level. Data points represent mean values with numbers of experimental replicates from four (30 °C) or five (20 °C) animals indicated in parentheses. Error bars in all plots represent SEM. Significance between pairs of data was tested with a two-sided Student's *t-*test. ***P<0.001, *P<0.05.

Na_V_1.9 channels are expressed at high levels in nociceptors and are proposed to influence excitability of these neurons by modulating their RMP^3^. To analyse the impact of mutation p.V1184A on the excitability of primary nociceptive neurons, we performed current-clamp recordings on small (<25 μm in diameter) isolated DRG neurons from wild-type mice transfected with constructs encoding either human wild-type or Na_V_1.9-V1184A mutant channels. To assess the temperature sensitivity of DRG neuron excitability, data were collected at 30 °C (Fig. 4a) and 20 °C (Fig. 4b).

When overexpressed in DRG neurons, p.V1184A mutant channels depolarized the cell membrane at rest (RMP) by 5.8 mV (*P*<0.05, two-tailed *t*-test) and 5.2 mV (*P*<0.05, two-tailed *t*-test) at 30 and 20 °C, respectively, when compared with cells overexpressing Na_V_1.9 wild type (Fig. 4c). In addition, mutant channels increased the action potential after-hyperpolarization voltage (*V*_min_) at 30 °C by 4.4 mV (*P*<0.05, two-tailed *t*-test) while they did not influence the after-hyperpolarization voltage at 20 °C. Other parameters characterizing the shapes of single evoked action potentials, that is, the voltage threshold for action potential firing (*V*_th_), the action potential peak voltage (*V*_max_), the action potential duration (Width), as well as the time constant describing the relaxation of the action potential after-hyperpolarization back to the resting level (*τ*_AHP_) were—at both temperatures—not significantly different between cells expressing Na_V_1.9 or Na_V_1.9-V1184A (Fig. 4c; *P*>0.05, two-tailed *t*-test). However, lowering the temperature from 30 to 20 °C increased the duration of action potentials by factors of 1.92±0.32 (*P*<0.001, two-tailed *t*-test) and 1.52±0.36 (*P*<0.001, two-tailed *t*-test) when cells were transfected with Na_V_1.9 and Na_V_1.9-V1184A, respectively. Temperature-dependent changes were furthermore observed for the relaxation time constant *τ*_AHP_, although statistically significant only for neurons expressing Na_V_1.9-V1184A (*P*<0.05, two-tailed *t*-test).

**Figure 5:**
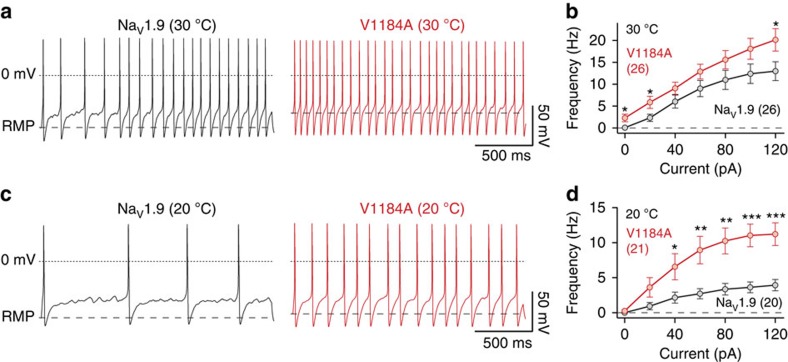
Na_V_1.9-V1184A channels cause hyperexcitability and reduced cold-sensitivity of DRG neurons. (**a**) Representative trains of action potentials at 30 °C, recorded from murine DRG neurons transfected with wild-type Na_V_1.9 (black) or mutant Na_V_1.9-V1184A (red), in response to 2 s current injections of 60 pA. Dotted lines mark 0 mV; dashed lines indicate the RMP. (**b**) Action potential frequencies obtained from experiments as shown in **a** as a function of the injected current. (**c**,**d**) Equivalent set of experiments as shown in **a** and **b**, performed at 20 °C. Data points represent mean values obtained from four (30 °C) or five (20 °C) animals with numbers of experimental replicates indicated in parentheses. Significance between pairs of data was tested with a two-sided Student's *t-*test. ***P<0.001, **P<0.01, *P<0.05.

Firing rates of neurons were determined by evoking repetitive action potentials with escalating current injections for periods of two seconds. Representative trains of action potentials recorded from neurons transfected with either human Na_V_1.9 or Na_V_1.9-V1184A at 30 and 20 °C are shown in Fig. 5a,c, respectively. Systematic assessment of action potential firing as a function of injected current revealed that neurons transfected with Na_V_1.9-V1184A fired—at either temperature—more action potentials than neurons transfected with wild-type Na_V_1.9, demonstrating pro-excitatory properties of Na_V_1.9-V1184A channels (Fig. 5b,d). Interestingly, cooling from 30 to 20 °C reduced the firing frequency of neurons expressing wild-type Na_V_1.9 by a factor of 3.2±0.1 in contrast to a factor of 1.6±0.1 (*P*<0.001, two-tailed *t*-test) for mutant p.V1184A (Fig. 5b,d). Thus, attenuation of DRG neuron excitability by low temperature is diminished in the presence of Na_V_1.9-V1184A, which is in line with the temperature dependence of the clinical symptoms.

### Comparison of mutation p.V1184A with p.L811P

**Figure 6:**
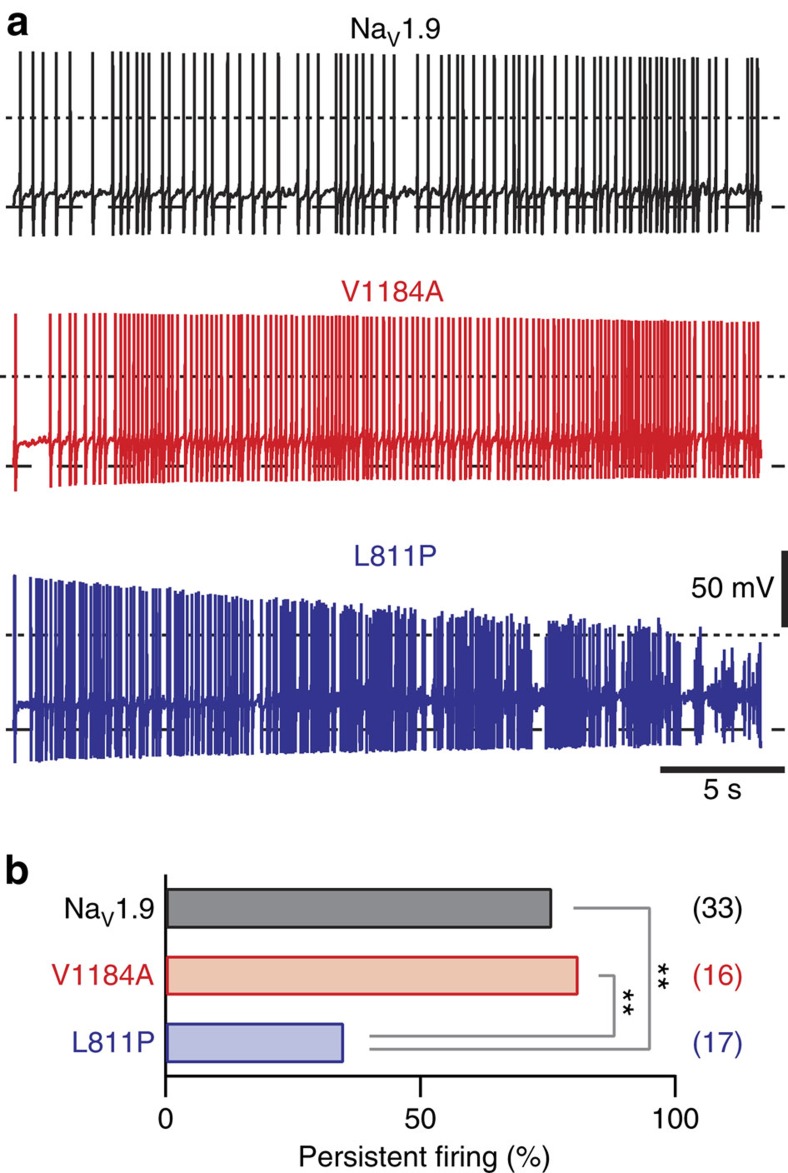
Na_V_1.9-V1184A and Na_V_1.9-L811P result in distinguishable firing characteristics of DRG neurons. (**a**) Representative trains of action potentials at 30 °C, recorded from murine DRG neurons transfected with Na_V_1.9 (black), Na_V_1.9-V1184A (red) or Na_V_1.9-L811P (blue), in response to 30 s current injections of 40 pA. Dotted lines mark 0 mV; dashed lines indicate the RMP. (**b**) Trains of action potentials as shown in **a**, obtained from repetitively firing neurons, were analysed to determine the fraction of neurons that maintained stable action potential amplitudes over 30 s. The criterion for ‘persistent firing' was defined as a reduction of peak action potential amplitude (*V*_max_) by <10 mV throughout the train. Numbers of experimental replicates are given in parentheses. Significance was tested with a *Z*-test. **P<0.01.

The phenotypes associated with mutation p.V1184A in Na_V_1.9 as well as those with mutations in Na_V_1.9 causing neuropathic pain conditions^17^,^18^,^19^ are in marked contrast to that of the Na_V_1.9 gain-of-function mutation p.L811P, which results in pain insensitivity^15^,^16^. The basis of this fundamental difference in the clinical outcome remained elusive. Therefore, we analysed the impact of p.L811P on excitability of DRG neurons under identical experimental conditions as used for p.V1184A. As illustrated in Supplementary Fig. 1, the RMP of neurons expressing p.L811P was increased by 5.9±1.4 mV (*P*<0.01, two-tailed *t*-test) at 30 °C and by 5.3±1.0 mV (*P*<0.01, two-tailed *t*-test) at 20 °C compared with neurons expressing wild-type Na_V_1.9. Parameters characterizing the shape of single evoked action potentials were only marginally affected by p.L811P (Supplementary Fig. 1c). Analysis of repetitive firing, evoked with current injections for 2 s, revealed that p.L811P rendered neurons hyperexcitable at both temperatures tested. Thus, this experimental protocol did not reveal overt differences between excitability of neurons transfected with p.L811P or p.V1184A. However, longer trains of action potentials revealed clearly distinguishable firing patterns between neurons expressing p.L811P or p.V1184A (Fig. 6). As illustrated in Fig. 6a,b, 76% of repetitively firing neurons transfected with wild-type Na_V_1.9 and 81% of repetitively firing neurons expressing p.V1184A maintained stable action potential amplitudes; that is, *V*_max_ diminished by <10 mV throughout the 30-s stimulus of 40 pA current injection, while the same criterion was fulfilled for only 35% of neurons expressing p.L811P (*P*<0.01, *Z*-test). Thus, p.L811P channels increase the probability of cumulative loss of action potential integrity in DRG neurons during extended firing periods.

## Discussion

Our study identified a heterozygous missense mutation (p.V1184A) in Na_V_1.9 as a cause for early onset cold-aggravated episodic peripheral pain. The mutation segregated with the disease phenotype in the affected family and showed slightly variable expressivity. Anti-inflammatory medication reduced the frequency of painful events, which was highest in childhood and gradually decreased with age. Similar age-dependent reduction of painful events as well as beneficial effects of anti-inflammatory drugs were reported for patients from two pedigrees suffering from Na_V_1.9-associated episodic peripheral pain^19^. The pain-alleviating effects of the anti-inflammatory therapy correlate with an observed upregulation of Na_V_1.9 under inflammatory conditions^2^,^23^,^24^ and emphasize a role of Na_V_1.9 in mediating inflammatory pain.

**Figure 7:**
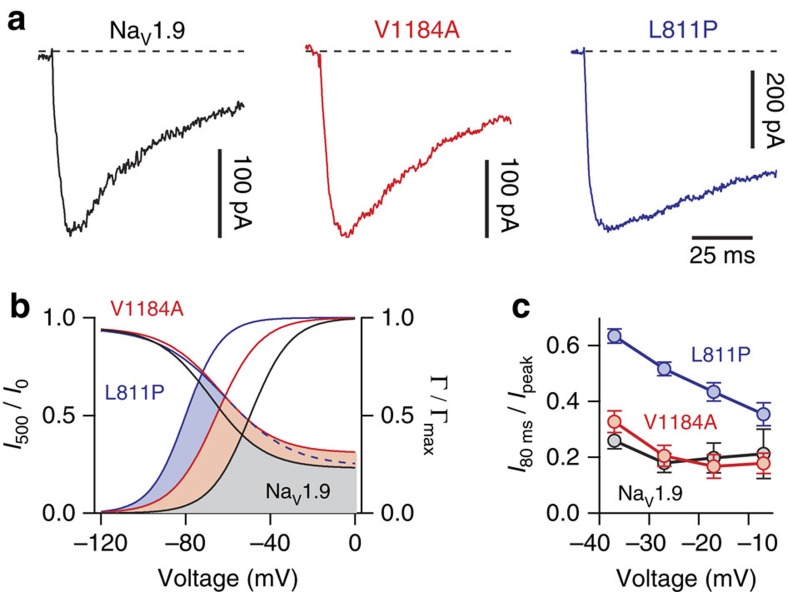
Different states of Na_V_1.9 hyperactivity. (**a**) Representative whole-cell current traces recorded at −37 mV from ND7/23 cells transiently expressing Na_V_1.9 (black), Na_V_1.9-V1184A (red) or Na_V_1.9-L811P (blue). Fast inactivation of Na_V_1.9 is not affected by mutation p.V1184A while mutation p.L811P slows down channel inactivation, thus prolonging channel opening. (**b**) Boltzmann functions describing the voltage dependences of opening (*Γ*/*Γ*_max_) and inactivation (*I*_500_/*I*_0_) of wild-type Na_V_1.9 (black) and mutants L811P (blue) and V1184A (red). Coloured areas indicate overlap between activation and inactivation curves, which is expected to result in a pro-excitatory window current; this window current is largest for mutation p.L811P followed by p.V1184A and wild-type Na_V_1.9. (**c**) Current traces as shown in **a** were analysed to determine the fraction of channels not inactivated 80 ms after triggering channel activation with various depolarizing voltage steps. Notably, fast inactivation of mutant p.L811P (*n*=11) is impaired in the entire voltage range while mutation p.V1184A (*n*=9) does not affect fast inactivation (*n*=10 for Na_V_1.9). Data points represent mean values with error bars indicating s.e.m. Data from Na_V_1.9-L811P as in Leipold *et al.* (2013).

We demonstrated that mutation p.V1184A confers gain-of-function attributes to Na_V_1.9 channels primarily by a hyperpolarizing shift in the voltage dependence of channel activation and a smaller voltage dependence of steady-state fast inactivation. The resulting increase in overlap of activation and inactivation of mutated channels predicts a larger steady-state window current^25^, which is expected to depolarize nociceptors resulting in hyperexcitability^2^,^3^,^24^ (Fig. 7b). On the basis of the models of K_V_ and Na_V_ channel structures^26^,^27^,^28^,^29^, mutation p.V1184A is located within a conserved sequence at the intracellular end of transmembrane segment 5 in domain III of Na_V_1.9 (Fig. 1c), a region highly relevant for transferring voltage-sensor movements to the channel pore^30^. Mutations in this region are expected to interfere with voltage-dependent channel gating.

Furthermore, Na_V_1.9-V1184A channels diminished the RMP in isolated small-diameter DRG neurons by >5 mV and increased the firing rate of nociceptive neurons in response to standardized current injections. This observation is consistent with previous studies showing similar pro-excitatory properties of gain-of-function variants of Na_V_1.9 linked to familial episodic pain^19^ and painful neuropathy^17^,^18^. Because low ambient temperature can trigger painful episodes in carriers of the p.V1184A mutation, we analysed the effect of temperature changes on the electrical properties of DRG neurons. Interestingly, cooling from 30 to 20 °C attenuated the excitability of neurons expressing Na_V_1.9-V1184A much less compared with neurons expressing wild-type Na_V_1.9, demonstrating that the relative impact of Na_V_1.9-V1184A on nociceptor excitability is larger at low temperature. We further showed that the mutation p.V1184A did not affect the Q_10_ values of the gating processes of Na_V_1.9, indicating that a cool environment does not *per se* augment the activity of Na_V_1.9-V1184A relative to the wild type.

The cold-aggravated pain symptoms in humans as well as the reduced attenuation of excitability of nociceptors expressing Na_V_1.9-V1184A are in line with a previous report showing that Na_V_1.9 is a key determinant of rodent cold-pain sensation^31^. Using Na_V_1.9-deficient animals, Lolignier *et al.*^31^ demonstrated that Na_V_1.9 channels are functionally upregulated specifically in cold-sensitive nociceptors and are required for normal cold-pain sensation. The authors proposed a model in which Na_V_1.9 in nociceptors serves as amplifier of signals generated by an as yet unknown cold transducer. This hypothesis is appealing because Na_V_1.9 is expressed not only in the cell bodies of nociceptors^32^,^33^ but also in the corresponding nerve terminals in the skin^34^, which express temperature-sensitive transducer proteins and are exposed to large variations in environmental temperatures. Accordingly, Na_V_1.9-V1184A channels may confer hypersensitivity to cold-responsive nociceptors because of overamplification of cold transducers. Such a scenario would be in line with our data showing that p.V1184A does not affect the intrinsic temperature dependence of Na_V_1.9.

The clinical symptoms caused by mutation p.V1184A in Na_V_1.9 are reminiscent of primary erythromelalgia, which is attributed to gain-of-function mutations of Na_V_1.7 channels^35^. Both conditions are characterized by episodic pain attacks in the lower and upper body extremities but seem to differ with respect to the temperature sensitivity of pain sensation. Specifically, p.V1184A-related pain is mostly aggravated by cooling and partly relieved by warming while primary erythromelalgia-associated pain is augmented by warming and relieved by cooling^5^. This is best illustrated by comparing the Na_V_1.9-V1184A-related disease with a primary erythromelalgia phenotype caused by the homologous mutation p.V1316A in Na_V_1.7 (ref. 10): p.V1316A in Na_V_1.7 causes warmth-aggravated severe burning pain in the body extremities, erythema in the affected body regions, and pain relief on cooling. A hallmark of Na_V_1.7 mutations linked to primary erythromelalgia—including p.V1316A—is a hyperpolarizing shift in the voltage dependence of channel activation with only minor impairment of channel inactivation^5^. As demonstrated here, the homologous mutation p.V1184A alters the properties of Na_V_1.9 channels in a very similar manner but instead results in a cooling-aggravated and thus a clinically distinguishable phenotype. Compatible with our report on the Na_V_1.9-V1184A-induced clinical symptoms, Huang *et al.*^18^ have reported low ambient temperature as a trigger of pain episodes in a patient suffering from a similar episodic peripheral pain phenotype caused by mutation p.I381T in Na_V_1.9. Likewise, Zhang *et al.*^19^ reported pain amelioration on warming of affected body areas in patients carrying Na_V_1.9 mutation p.R225C or p.A808G. Notably, Han *et al.*^36^ described mutation p.G699R, located in the S4/S5 linker in domain II of Na_V_1.9, which causes painful peripheral neuropathy with a temperature dependence reminiscent of erythromelalgia; that is, in this case, pain episodes are aggravated by warmth and relieved by cooling.

The gain-of-function mutations in Na_V_1.9 reported previously^17^,^18^,^19^, as well as this report on mutation p.V1184A are in marked contrast to the Na_V_1.9 gain-of-function mutation p.L811P that results in pain insensitivity^15^,^16^. The basis of this fundamental difference in the clinical outcome remained elusive. To infer potential differences between p.V1184A and p.L811P we assessed the functions of both mutations under identical experimental conditions (Figs 6 and 7). Both mutations affect amino acids on the intracellular face of the channel pore in domains II and III (Fig. 1c)—regions well-known to be involved in the gating of Na_V_ channels^37^. A comparison of current responses revealed substantial differences with respect to fast channel inactivation (Fig. 7a,c), a process largely mediated by the interaction of a hydrophobic IFM (Isoleucine–Phenylalanine–Methionine) motif in the loop connecting channel domains III and IV with the intracellular face of the pore (Fig. 1c). Interestingly, fast inactivation kinetics of Na_V_1.9-V1184A channels are almost indistinguishable from inactivation of the wild type, whereas mutation p.L811P causes profound slowing of channel inactivation, thus augmenting Na^+^ influx through Na_V_1.9 (ref. 15). In addition, both mutations marginally affect the voltage dependence of channel inactivation. However, p.L811P shifts the voltage dependence of opening of Na_V_1.9 by −29 mV, whereas mutation p.V1184A shifts the same parameter by only −20 mV, clearly demonstrating that mutation p.L811P facilitates opening of Na_V_1.9 more strongly than mutation p.V1184A (Fig. 7b). As a result, the window current generated by the overlap of activation and inactivation—here used as an operational indicator of channel activity—is largest for mutation p.L811P followed by p.V1184A and wild-type Na_V_1.9 (Fig. 7b). These data demonstrate that both mutations confer quantitatively and qualitatively different gain-of-function properties to Na_V_1.9.

Consistent with the gain-of-function on the channel level, both Na_V_1.9-V1184A and Na_V_1.9-L811P increased the action potential firing frequency of DRG neurons on stimulation with short current injections for 2 s (Fig. 5 and Supplementary Fig. 1d–g). Thus, neurons expressing either p.V1184A or p.L811P must be considered hyperexcitable under these experimental conditions. Intriguingly, analysis of extended periods of repetitive firing revealed that, in contrast to p.V1184A, mutation p.L811P leads to a substantial instability of DRG neuron excitability; that is, the probability of progressive loss of action potential amplitude is increased in a substantial portion of neurons expressing p.L811P mutant channels (Fig. 6). This instability may be caused by increased influx of Na^+^ through p.L811P as it is predicted by the larger window current (Fig. 7b) and the slower inactivation kinetics of p.L811P compared with p.V1184A or wild-type Na_V_1.9 (Fig. 7a). Excessive Na^+^ influx during longer periods of action potential firing may therefore lead to a conduction block in the transfected neurons. However, chronic Na_V_1.9 dysfunction in patients will additionally result in secondary effects, including altered gene expression and changes in the synaptic plasticity. In summary, the missense change p.V1184A, which causes cold-aggravated peripheral pain in humans, leads to higher open probability of mutated Na_V_1.9 channels and to hyperexcitability of DRG neurons; this hyperexcitability is less attenuated at low ambient temperature compared with wild-type neurons. The degree and quality of functional change on the level of the ion channel, however, differs from the gain-of-function mutation p.L811P; the latter only transiently results in DRG neuron hyperexcitability and ultimately leads to loss-of-function and pain insensitivity. Correlations between a clinical pain phenotype and Na_V_1.9 channel function thus require a very detailed analysis of the channel's gating parameters where the exact relationship of voltage-dependent channel activation and inactivation appears to be decisive for the overall outcome.

## Methods

### Subjects

The study was approved by the local research ethics committee or institutional review board of the participating institutions. Informed consent was obtained from all participating family members.

### Whole-exome sequencing

For whole-exome sequencing, 1 μg of DNA from two family members with episodic pain was fragmented using sonication technology (Covaris, Woburn, MA, USA). The fragments were end-repaired and adaptor-ligated including incorporation of sample index barcodes. After size selection, the libraries were subjected to an enrichment process (NimbleGen SeqCap EZ Human Exome Library v2.0, Roche NimbleGen). Samples were sequenced on an Illumina HiSeq 2000 sequencing instrument. This resulted in 6.6 (individual 1) and 6.4 (individual 2) Gb of mapped sequences with a mean coverage of 77/78 and a 30 × coverage of 78/84% and a 10 × coverage of 95.8/96.4% of target sequences. For data analysis, the Varbank pipeline v.2.3 and interface was used (https://anubis.ccg.uni-koeln.de/varbank/). Primary data were filtered according to signal purity with the illumina real-time analysis software v1.8. Subsequently, the reads were mapped to the human genome reference build hg19 using the Burrows-Wheeler alignment algorithm. GATK v.1.6 was used to mark duplicated reads, to perform a local realignment around short insertion and deletions, to recalibrate the base quality scores, and to call single nucleotide polymorphisms and short indels. Subsequent Sanger sequencing of *SCN11A* exons was performed using standard procedures. Primer sequences are available on request.

### Skin biopsies

Punch biopsies (3 mm) were taken from the volar forearm. The biopsies were fixed with Zamboni solution for immunohistochemistry and with 3.9% buffered glutaraldehyde for electron microscopy. For immunohistochemistry 40 μm cryostat sections were prepared and stained with anti-PGP9.5 primary antibody at 1:100 dilution (DCS-Diagnostics, catalogue number PI647C01), and Alexa Fluor 488-labelled goat anti-rabbit secondary antibody at 1:2,000 dilution (Thermo-Fischer, catalogue number A-11008)^38^. Intra-epidermal nerve fiber density was quantified following the second set of counting rules^39^, counting both fibres crossing the epidermal basement membrane and isolated nerve fragments in the epidermis that did not cross the basement membrane^38^. These counting rules were followed because they had been used by the group that had established the reference values for distal forearm skin innervation^20^. The glutaraldehyde-fixed tissues were post-fixed with 1% OsO_4_ in 0.1 M cacodylate buffer and embedded in epoxy resin. Ultrathin sections, after contrast enhancement with uranyl acetate and lead citrate, were examined under a Philips EM 400 T electron microscope. Images were taken with an Olympus Veleta camera system.

### cDNA constructs for human Na_V_1.9 and mutants

Human Na_V_1.9 cDNA (NM_014139.2) in pcDNA3.1 was used as template^15^. The c.3551T>C (p.Val1184Ala) mutation was introduced by PCR-based mutagenesis and replaced a cDNA fragment flanked by *Blp*I on both sites. Correct orientation of the *Blp*I-fragment was confirmed, and the coding sequence of *SCN11A* was verified by Sanger sequencing.

### Isolation and transfection of mouse DRGs

Animal care and experimental procedures followed the guidelines established by the animal welfare committee of the University of Jena. For action potential recordings, DRG from all levels of the spinal cord of 6–11-weeks-old wild-type C57BL6 mice were extracted and processed as described^40^. Isolated DRG neurons were transfected by electroporation using a 4D-Nucleofector (Lonza, Basel, Switzerland) with the P3 Primary Cell 4D-Nucleofector X Kit S (V4XP-3032). Briefly, DRG neurons from each animal were split in two equal lots to enable experiments with wild-type and mutant Na_V_1.9 channels using the same batch of cells. After centrifugation (100 g for 3 min) both cell pellets were resuspended individually in 20 μl of P3 primary cell solution containing supplement 1 and 0.3 μg of a plasmid encoding the enhanced green fluorescent protein. Subsequently, one lot was supplemented with 1.2 μg of a Na_V_1.9-encoding plasmid, while the second lot was supplemented with 1.2 μg of a vector encoding mutant Na_V_1.9 variants. Transfection was performed using the electroporation protocol CA137 of the 4D-Neucleofector. After electroporation, 150 μl of low calcium Roswell Park Memorial Institute (Invitrogen) 1640 medium was added to each cell suspension and cells were allowed to recover for 10 min in a 10% CO_2_ incubator at 37 °C. The cell suspensions were then diluted in 300 μl DRG medium and immediately seeded on poly-D-lysin/laminin-coated glass coverslips, which were placed in the slots of a 24-well plate containing 1 ml of DRG medium per well. DRG medium contained 89.5% DMEM/F12 (Dulbecco's Modified Eagles Medium with Ham's F12; Invitrogen) supplemented with 9.5% fetal calf serum and 1% penicillin/streptomycin (Invitrogen). Transfected cells were incubated in a 10% CO_2_ incubator at 37 °C and used for electrophysiological experiments 15–24 h after transfection. Current-clamp recordings were restricted to successfully transfected small-diameter (<25 μm) DRG neurons, which were identified visually by their green florescence using a 50 W HBO lamp as light source and a green fluorescent protein filter set.

### Cell culture and transfection

ND7/23 cells—a hybrid of mouse neuroblastoma and rat DRG neurons—, were maintained in 90% DMEM supplemented with 10% fetal calf serum in a 10% CO_2_ incubator at 37 °C. Cells were trypsinized, diluted with culture medium, and grown in 35 mm dishes. When grown to 30–50% confluence, cells were transfected with a 5:1 ratio of a DNA plasmid encoding either Na_V_1.9 or Na_V_1.9 mutants and a vector encoding the CD8 antigen using the Rotifect transfection kit (Carl Roth, Karlsruhe, Germany) as described earlier^15^. Transfected cells were maintained in a 10% CO_2_ incubator at 28 °C for up to 12 h. Voltage-clamp recordings were performed after an additional 1 h recovery period at 37 °C. Anti-CD8-coated Dynabeads (Deutsche Dynal GmbH, Hamburg, Germany) were used for visual identification of individual transfected cells.

### Electrophysiology

Current and voltage recordings were obtained in the whole-cell configuration of the patch-clamp method using an EPC-10 patch-clamp amplifier operated by PatchMaster software (HEKA Elektronik, Lambrecht, Germany). Patch pipettes were fabricated from Kimax borosilicate glass of about 1.0–2.5 MΩ resistance and coated with room temperature vulcanization silicone adhesive (Dow Corning GmbH, Wiesbaden Germany) to reduce tip capacitance. Series resistance was corrected electronically up to 85% and all voltages were corrected for the liquid junction potential. Bath solution for current-clamp recordings contained (in mM) 120 NaCl, 3 KCl, 2.5 CaCl_2_, 1 MgCl_2_, 30 HEPES, 15 glucose (pH 7.4 with NaOH), and the pipette 125 KCl, 8 NaCl, 1 CaCl_2_, 1 MgCl_2_, 0.4 Na_2_-GTP, 4 Mg-ATP, 10 EGTA, 10 HEPES (pH 7.3 with KOH). Bath solution for voltage-clamp recordings contained (in mM) 150 NaCl, 2 KCl, 1.5 CaCl_2_, 1 MgCl_2_, 10 HEPES (pH 7.4 with NaOH) and was supplemented with 0.0003 tetrodotoxin to block endogenous Na^+^ currents in ND7/23 cells; the pipette contained (in mM) 35 NaCl, 105 CsF, 10 EGTA, 10 HEPES (pH 7.3 with CsOH). Temperature was controlled by continuous perfusion of cells with tempered bath solution using a SHM-8 in-line solution heater feedback-controlled by a TC-324B temperature controller (both Warner Instruments, Hamden, USA).

*Current-clamp recordings*. The resting membrane voltage was measured by zero current injection directly after establishing the whole-cell configuration. Single action potentials were evoked at 30 or 20 °C by injecting a current of 100–200 pA for a period of 5 or 10 ms, respectively, followed by a 200-ms pulse without current injection. Sampling interval for voltage measurements was 50 μs. The voltage threshold of action potential firing, *V*_th_, was defined as voltage at which d*V*/d*t* reached the level of 0.03 × (d*V*/d*t*_max_−d*V*/d*t*_min_)+d*V*/d*t*_min_. Trains of action potentials were evoked repetitively by 2 s current injections increasing in steps of 20 pA.

*Voltage-clamp recordings*. In voltage-clamp experiments with ND7/23 cells the holding potential was set to –137 mV. Data were low-pass filtered at 5 kHz and digitized with a sampling interval of 40 μs. Leak and capacitive currents were measured at –117 and –127 mV and subtracted manually.

*Channel activation*. To measure channel activation, test depolarizations between –127 and 23 mV were applied in steps of 10 mV every 3 s. The voltage dependence of channel activation was estimated from mean current densities using the following formalism:


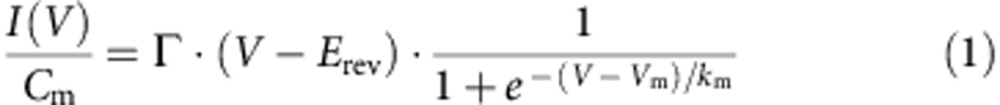


with the cell capacitance *C*_m_, conductance density (Γ), and the reversal potential (*E*_rev_). *V*_m_ is the half-maximal activation voltage and *k*_m_ the corresponding slope factor.

*Gating kinetics*. The kinetics of activation and fast inactivation of Na_V_1.9 and Na_V_1.9-V1184A channels were analysed between –67 and –7 mV with a model assuming one activation gate and one inactivation gate:


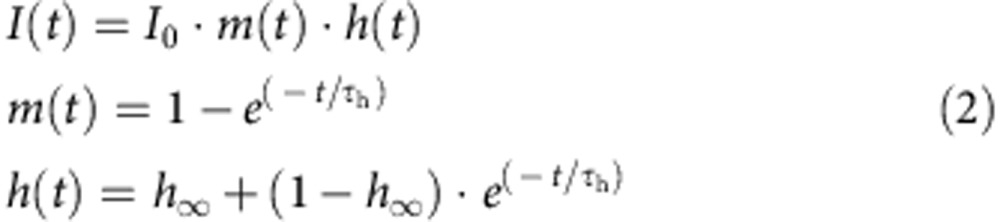


with the current amplitude *I*_0_. *m*(*t*) and *h*(*t*) describe the kinetics of channel activation and inactivation, respectively; *τ*_m_ and *τ*_h_ are the associated time constants, and *h*_∞_ characterizes the fraction of non-inactivating current after infinite time.

*Steady-state inactivation*. Na_V_1.9 channels were activated with a first 50 ms test pulse to –47 mV followed by a conditioning interval of 500 ms at voltages ranging from –127 to –17 mV in steps of 10 mV. Peak currents of not inactivated channels were measured in a subsequent 50 ms test pulse. The repetition interval was 20 s. The current amplitude after conditioning (*I*_500 ms_) normalized to the control current amplitude before conditioning (*I*_0_) was described with the following Boltzmann formalism:


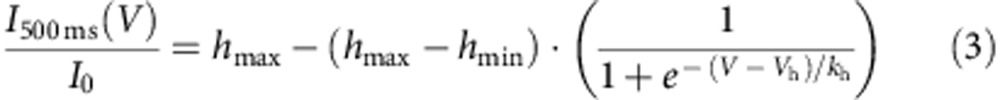


with the maximal and minimal channel availability *h*_max_ and *h*_min_, the half-maximal inactivation voltage *V*_h_ and the corresponding slope factor *k*_h_.

*Channel deactivation*. Channels were first activated with a 25-ms depolarization to −47 mV. Subsequently, deactivation was triggered with a 150-ms repolarization period at voltages ranging from –117 to –77 mV in steps of 5 mV with a repetition interval of 10 s. The current decay during the repolarization period was fitted according to:


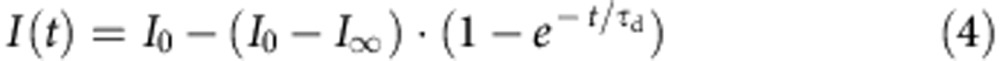


where *I*_0_ is the maximal current amplitude and *I*_*∞*_ the current remaining after infinite time. *τ*_d_ is the deactivation time constant. The voltage dependence of activation and deactivation time constants was described with a formalism assuming a one-step process for channel opening and closing:


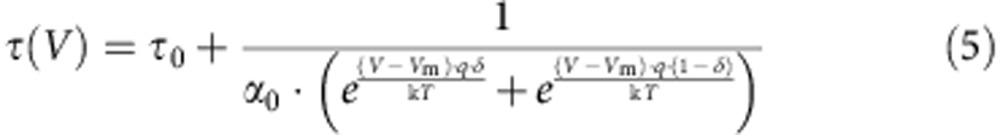


*τ*_0_ is the voltage-independent, limiting speed of deactivation, and *α*_0_ is the rate at the equilibrium voltage *V*_m_, and k*T* is the thermal energy. *q* is the total gating charge transfer and *δ* is the symmetry factor specifying the gating charge fraction associated to channel activation.

*Temperature dependence*. *Q*_10_ temperature coefficients, specifying the change of reaction rates in response to a temperature change of 10 °C were calculated according to:


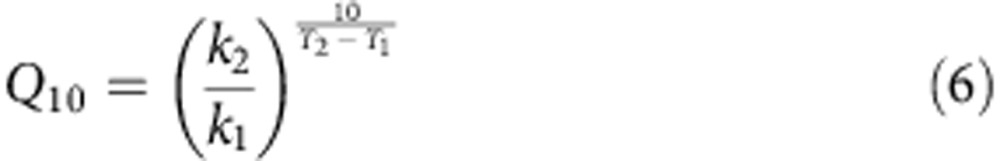


where *k*_1_ and *k*_2_ are the reaction rates obtained at temperatures *T*_1_ and *T*_2_, respectively.

Data were analysed with FitMaster (HEKA Elektronik) and IgorPro (WaveMetrics, Lake Oswego, OR, USA) software. Data are presented as mean±s.e.m., (*n*) with *n* being the number of independent experiments. Statistical comparisons of two groups of data were made using the two-tailed Student's *t*-test or the *Z*-Test; *P* values are given explicitly when appropriate.

## Additional information

**How to cite this article:** Leipold, E. *et al.* Cold-aggravated pain in humans caused by a hyperactive NaV1.9 channel mutant. *Nat. Commun.* 6:10049 doi: 10.1038/ncomms10049 (2015).

## Supplementary Material

Supplementary Information Supplementary Figure 1

## Figures and Tables

**Table 1:** Clinical phenotype of the family

**Symptoms**	**I.1**	**II.1**	**II.3**	**III.2**	**III.4**
*Pain*					
LocationSkin changes	Lower extremities, occasionally upper. Starts in joints (ankles, knees, elbows, wrists only) and radiates to arms and legsOccasional hives	Lower extremities, less commonly upper extremities. Starts in joints and radiates into arms or legsUnknown	Lower extremities, occasionally upper. Starts in joints and radiates to arms and legsOccasional flushing of neck and chest when young	Lower extremities, less commonly upper extremities. Starts in joints and radiates into arms or legsUnknown	Lower extremities, occasionally upper. Starts in joints and radiates to arms and legsOccasional flushing of neck and face, and sweating
Onset	Infancy. Pain episodes can occur any time	Unknown	Since age 18 months. Pain episodes can occur any time but more at night	Infancy	Since age 10 months. Pain episodes often occur late afternoon/early evening
Triggers	Gluten	Cold temperature	Gluten‘When there is stress on the body, such as exhaustion and illness'	None reported	Cold temperature, gluten, nitrates‘When there is stress on the body, such as exhaustion and illness'
Frequency	2–3 times/month with lifestyle modifications and medication	2–3 times/month	2–3 times/month with lifestyle modifications and medication	2–3 times/month	∼60 times/month if no treatment.2–3 times/month with lifestyle modifications and medication
Duration	If no medication, cycling between 20 min of pain and 20 min without pain for 3 h	20–30 min	20–30 min	20–30 min	20–30 min
Responsiveness to medication	Ibuprofen prophylactically	Ibuprofen	Ibuprofen	IbuprofenNaproxen	IbuprofenNaproxenColchicine
Consequence	Impairs desire to walk and fine motor manipulationDesire to keep arms and legs in certain positions and move them in certain ways to alleviate pain	Walking uncomfortable	Impairs desire to walk and fine motor manipulationDesire to keep arms and legs in certain positions and move them in certain ways to alleviate pain	Walking uncomfortable	Impairs desire to walk and fine motor manipulationDesire to keep arms and legs in certain positions and move them in certain ways to alleviate pain
*Skin*					
Skin injuries caused by inability to feel pain	N	N	N	N	N
Prolonged healing	N	N	N	N	N
*Cold/heat tolerance*					
Problem tolerating heat/cold	N	Sensitive to cold; pain somewhat eased by warmth	N	N	Sensitive to cold. Cold can cause leg pain
Excess/insufficient sweating	N	N	N	N	N
*Development*					
Failure to thrive	N	N	N	N	N
Intellectual disability	N	N	N	N	N
Motor delay	N	N	N	N	N
*Gastrointestinal*					
Constipation	Yes, since age 18 years	N	Yes, since age 18 years	N	N
Diarrhoea	Yes, since age 18 years	N	Severe pain causes diarrhoea	N	Severe pain causes diarrhoea
Pain passing stool	N	N	N	N	N
*Psychology*					
Psychological, emotional problems	N	N	N	N	N
*Musculoskeletal*					
Joint issues	N	N	N	N	N
Muscle weakness	N	N	N	N	N

N = not reported. Phenotype information was provided by I.1 and II.3.
