# Supplementary material for: Cold-aggravated pain in humans caused by a hyperactive NaV1.9 channel mutant
Source: Nat Commun. 2015 Dec 8;6:10049. doi: 10.1038/ncomms10049 (PMC4686659; doi:10.1038/ncomms10049)
Supplement: Supplementary Information — Supplementary Figure 1 [file ncomms10049-s1.pdf]

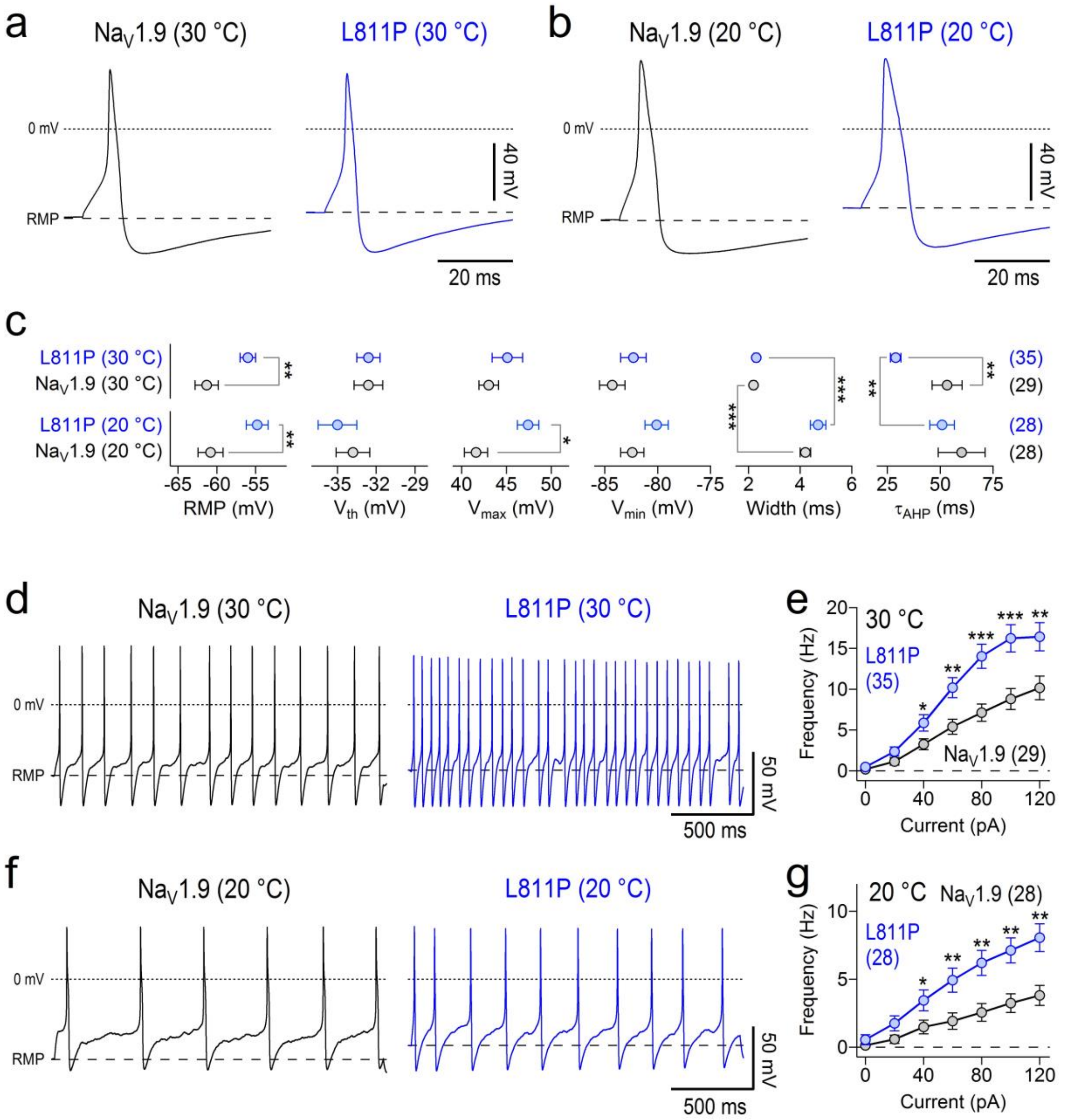

Supplementary Figure 1 | Na_V1.9-L811P channels depolarize DRG neurons and increase action potential firing frequencies. Representative single action potentials at 30 °C (**a**) or 20 °C (**b**), elicited in murine DRG neurons transfected with Na_V1.9 (black) or mutant Na_V1.9-L811P (blue) in response to current injections of 200 pA for 5 ms (a) or 10 ms (b). Dotted lines indicate 0 mV; dashed lines mark levels of the resting membrane potential (RMP). (**c**) Parameters characterizing action potential properties. RMP: resting membrane potential; V_{th}: action potential voltage threshold; V_{max}: maximum action potential voltage; V_{min}: minimum voltage during after-hyperpolarization; *Width*: action potential width at 0 mV; τ_{AHP}:

single-exponential time constant characterizing the relaxation of the membrane voltage from the action potential after-hyperpolarization (V_{min}) back to the resting level. (**d**) Representative trains of action potentials at 30 °C, recorded from murine DRG neurons transfected with $Na_V1.9$ (black) or mutant $Na_V1.9$-V1184A (red), in response to 2-s current injections of 60 pA. (**e**) Action potential frequencies obtained from experiments as shown in (d) as a function of the injected current. (**f**, **g**) Equivalent set of experiments as shown in (d) and (e), performed at 20 °C. Data points represent mean values obtained from 3 (30 °C) and 5 (20 °C) animals with numbers of independent experimental replicates in parentheses. Error bars in all plots represent SEM. Significance between data sets was tested with a two-sided Student's *t* test. *** $P < 0.001$, ** $P < 0.01$, * $P < 0.05$.
